# Targeting the proteostasis network in Huntington’s disease

**DOI:** 10.1016/j.arr.2018.11.006

**Published:** 2019-01

**Authors:** Tânia R. Soares, Sara D. Reis, Brígida R. Pinho, Michael R. Duchen, Jorge M.A. Oliveira

**Affiliations:** a REQUIMTE/LAQV, Department of Drug Sciences, Pharmacology Lab, Faculty of Pharmacy, University of Porto, 4050-313, Porto, Portugal; b Department of Cell and Developmental Biology, University College London, London, WC1E 6BT, UK; c Consortium for Mitochondrial Research (CfMR), University College London, Gower Street, WC1E 6BT, London, UK

**Keywords:** Huntington’s disease, Proteostasis, Mitochondria, Chaperones, Autophagy, Proteasome

## Abstract

Huntington’s disease (HD) is an autosomal dominant neurodegenerative disorder caused by a polyglutamine expansion mutation in the huntingtin protein. Expansions above 40 polyglutamine repeats are invariably fatal, following a symptomatic period characterised by choreiform movements, behavioural abnormalities, and cognitive decline. While mutant huntingtin (mHtt) is widely expressed from early life, most patients with HD present in mid-adulthood, highlighting the role of ageing in disease pathogenesis. mHtt undergoes proteolytic cleavage, misfolding, accumulation, and aggregation into inclusion bodies. The emerging model of HD pathogenesis proposes that the chronic production of misfolded mHtt overwhelms the chaperone machinery, diverting other misfolded clients to the proteasome and the autophagy pathways, ultimately leading to a global collapse of the proteostasis network. Multiple converging hypotheses also implicate ageing and its impact in the dysfunction of organelles as additional contributing factors to the collapse of proteostasis in HD. In particular, mitochondrial function is required to sustain the activity of ATP-dependent chaperones and proteolytic machinery. Recent studies elucidating mitochondria-endoplasmic reticulum interactions and uncovering a dedicated proteostasis machinery in mitochondria, suggest that mitochondria play a more active role in the maintenance of cellular proteostasis than previously thought. The enhancement of cytosolic proteostasis pathways shows promise for HD treatment, protecting cells from the detrimental effects of mHtt accumulation. In this review, we consider how mHtt and its post translational modifications interfere with protein quality control pathways, and how the pharmacological and genetic modulation of components of the proteostasis network impact disease phenotypes in cellular and *in vivo* HD models.

## Highlights

• Synergism between mutant huntingtin (mHtt) and ageing collapses proteostasis in HD.
• mHtt overwhelms chaperones, accumulating misfolded proteins that impair the UPS.
• mHtt disturbs the autophagy pathway *via* multiple pleiotropic effects.
• Mitochondria play an active role in the maintenance of cellular proteostasis.
• Modulating the proteostasis network impacts disease phenotypes in cells and *in vivo.*

## 1 Introduction

Huntington’s Disease (HD) is a neurodegenerative disease caused by a CAG repeat expansion mutation in the exon 1 of the huntingtin (Htt) gene. Mutant Htt (mHtt) thus contains an expanded polyglutamine (polyQ) tract in the N-terminal region, which is considered a main driver of mHtt proteotoxicity. HD has an estimated prevalence of 4–10 per 100,000 individuals in the Western world (Ross and Tabrizi, 2011), with worldwide variation surpassing tenfold differences between distinct geographical regions (Rawlins et al., 2016). Expansions of 40 or more polyQ are fully penetrant, and the greater the number of tandem repeats, the earlier is the age of onset (Squitieri and Jankovic, 2012). Despite the widespread mHtt expression, HD neurodegeneration is particularly severe in the striatum, where a combination of intrinsic vulnerability and non-cell autonomous factors preferentially kills GABAergic medium spiny neurons while sparing striatal interneurons (Ehrlich, 2012; Fu et al., 2018). Clinically, the disease is characterized by a progressive extra-pyramidal motor dysfunction, psychiatric disturbance and cognitive decline (Ross et al., 2014). Although mHtt is expressed throughout the life of the individual, HD onset typically occurs around the 4th decade of life. This suggests that there are compensatory mechanisms that limit mHtt proteotoxicity early in life, but that such mechanisms are eventually overtaken by ageing and disease progression (Arrasate and Finkbeiner, 2012).

The cellular proteome is maintained by the coordinated activity of a protein homeostasis (proteostasis) network, which regulates protein synthesis, folding, transport and degradation (for a recent review, see Labbadia and Morimoto, 2015). The proteostasis network can be induced in response to protein misfolding and aggregation, and comprises several quality control systems, such as molecular chaperones and proteolytic pathways (Hipp et al., 2014). Molecular chaperones assist protein folding and disaggregation (Hartl et al., 2011; Nillegoda et al., 2018). Chaperones of the heat shock protein (Hsp) 90 and Hsp70 families are the main effectors of the proteostasis network, acting together in a multiprotein complex that includes co-chaperones such as Hsp40 (J-protein) (Pratt et al., 2015). When misfolded proteins escape chaperone control they are usually targeted for degradation, either through the ubiquitin-proteasome system (UPS) or by autophagy (Hipp et al., 2014). The UPS is the main route of protein degradation in mammalian cells, acting in both cytoplasm and nucleus, whereas autophagy functions mainly in the cytoplasm (Hipp et al., 2014; Li and Li, 2011).

UPS-mediated degradation depends on the recognition of protein substrates that have been flagged through polyubiquitination (McKinnon and Tabrizi, 2014), a process catalysed by a triple enzymatic cascade, involving an E1 ubiquitin activator, an E2 conjugase and an E3 ligase (Dantuma and Bott, 2014). Polyubiquitinated proteins are recognized by the 26S proteasome for degradation. The 26S proteasome is an ATP-dependent proteolytic complex constituted by one or two 19S regulatory particles, and a 20S core particle. The regulatory particle recognizes, deubiquitinates and unfolds the substrate, which is then translocated to the cavity of the core particle, where multiple catalytic sites mediate its degradation (Li and Li, 2011; McKinnon and Tabrizi, 2014; VerPlank and Goldberg, 2017).

Autophagy-associated pathways for the clearance of cytosolic substrates converge on lysosomal degradation, and are broadly categorised as micro-, macro- and chaperone–mediated autophagy (CMA) (Cortes and La Spada, 2014). In microautophagy, cytosolic components are directly engulfed by the lysosome (Shpilka and Elazar, 2011). In CMA, soluble proteins are directly translocated into the lysosome after recognition of a KFERQ-like motif by the molecular chaperone Hsc70. In macroautophagy (hereafter referred to as autophagy) cytosolic substrates are engulfed by a double membrane structure, the autophagosome, which subsequently fuses with lysosomes (Cortes and La Spada, 2014). A more recent concept is that of selective autophagy, meaning the selective degradation of substrates recognized by specific autophagic receptors, such as p62 (Menzies et al., 2015; Wong and Holzbaur, 2015).

Data from HD patients and experimental models indicate that the accumulation and aggregation of mHtt are associated with a dysfunctional proteostasis network (Margulis and Finkbeiner, 2014; Sieradzan et al., 1999). A better understanding of the mechanisms by which mHtt interferes with protein quality control and degradation pathways, and how these pathways can be modulated, may offer new therapeutic avenues for HD. In the first section of this paper, we review the evidence for impaired proteostasis in HD. We consider the interplay between diffuse and aggregated mHtt, the stress imposed by such mHtt species on molecular chaperones and proteolytic pathways, and how this might synergise with ageing-associated changes in the proteostasis network. We address recent findings concerning impairments in autophagy and the potential contribution of a loss on normal Htt function. Moreover, we consider the emerging concept of mitochondrial proteostasis and how the dysfunction of this organelle in HD may impact the cellular proteostasis network. In the second section, we review recent data from HD models with genetic and pharmacological modulation of specific targets in the proteostasis network. We conclude with a discussion of the current model of impaired proteostasis in HD, how it might relate to selective neurodegeneration, and the implications for future directions of research in this field.

## 2 Section I - how is proteostasis impaired in HD?

### 2.1 The interplay of diffuse and aggregated mHtt

**Fig. 1:**
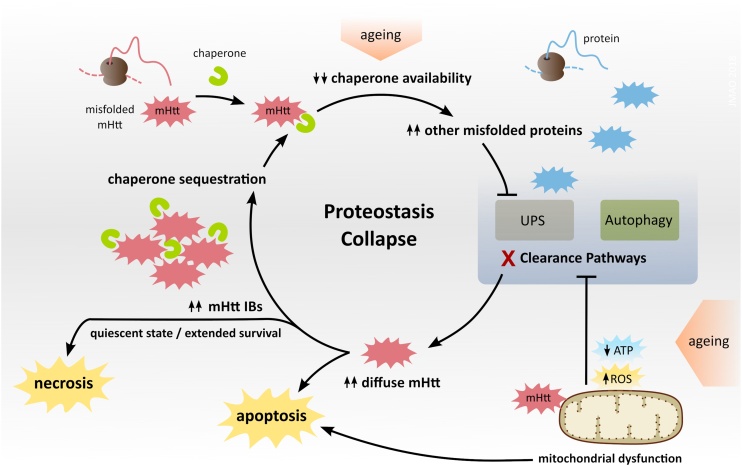
**Collapse of proteostasis in HD.** Chronic production of misfolded mHtt titrates chaperones, which acts synergistically with ageing through decreasing chaperone availability and thus leading to the misfolding and accumulation of other chaperone client proteins. The latter are diverted to the proteasome, exceeding its capacity, and causing UPS impairment (Hipp et al., 2014, 2012). Energy-dependent protein clearance pathways can be further impaired when ATP production is limited by mitochondrial dysfunction. Proteostasis collapse increases the levels of diffuse mHtt, which may trigger apoptosis or promote aggregation into inclusions bodies (IBs). By sequestering mHtt, IBs can extend survival, but they also recruit proteostasis components, in a vicious cycle that gradually disrupts cellular homeostasis, promoting a quiescent state that eventually culminates in necrosis (Ramdzan et al., 2017).

The aggregation of mHtt into inclusion bodies (IBs) is considered one of the central hallmarks of HD, although their role in HD pathogenesis remains incompletely understood (Margulis and Finkbeiner, 2014). The detection of IBs in cortical and striatal brain regions affected by HD (DiFiglia et al., 1997), as well as the observation of symptomatic rescue in HD mice treated with aggregation inhibitors, have supported the notion that IBs are toxic (Katsuno et al., 2004; Tanaka et al., 2004). However, the opposite notion that IBs are a coping response has been promoted by longitudinal survival analyses in cell models, which have revealed that the levels of diffuse mHtt predict death or IB formation, and that the latter decreases diffuse mHtt and increases survival (Arrasate et al., 2004). IB formation thus seems to reduce the levels of diffuse toxic species of mHtt (Arrasate et al., 2004), and also to rescue UPS impairment in HD cellular models (Mitra et al., 2009). Subsequently, it was proposed that diffuse mHtt might inhibit the proteasome indirectly, by saturating the protein folding machinery and diverting an excess of other misfolded/ubiquitinated proteins to the UPS that then becomes impaired (Bersuker et al., 2016; Hipp et al., 2012) (Fig. 1).

Several studies associate the toxicity of IBs with their ability to establish aberrant interactions with the proteome, and to sequester key cellular components such as proteostasis machinery (Gasset-Rosa et al., 2017; Park et al., 2013; Seidel et al., 2016). Hsp40 chaperones, which mediate the delivery of misfolded proteins to the nucleus for proteasomal degradation, were sequestered in IBs in yeast expressing mHtt and in brains of HD patients (Park et al., 2013; Seidel et al., 2016). Also, the constitutive Hsp70 (Hsc70), involved in protein folding, degradation, and prevention of aggregation, was sequestered in IBs in HD cell models (Yu et al., 2014). The toxicity of IBs also involves the physical disruption of cellular structures, as recently shown for the nuclear envelope (Gasset-Rosa et al., 2017) and ER membranes (Bauerlein et al., 2017). IBs in the cytosol obstruct intracellular trafficking (Chang et al., 2006), and were shown to impair the nucleocytoplasmic transport of proteins and RNA by sequestering proteins involved in nuclear transport (Woerner et al., 2016). Increases in nuclear IBs have also been correlated with increased cytotoxicity, as found in a study that depleted a key element of the ribosomal quality control (RQC) system (Zheng et al., 2017). Another study showed that RQC impairment promotes the occurrence of multiple small mHtt aggregates, instead of a single large aggregate, and that the former are more toxic and disrupt the actin cytoskeleton (Yang et al., 2016). Although further mechanistic studies are necessary, these studies suggest that altered activity of the RQC system may play an important role in mHtt aggregation and associated toxicity.

Thus, a consensus is emerging to suggest that mHtt aggregation into IBs plays a dual role, combining protective and toxic effects. Cells expressing high levels of diffuse mHtt are more likely to initiate apoptosis. Cells that form IBs show decreased levels of diffuse mHtt, but progressively convert into a quiescent state as the IBs also sequester other crucial proteins. While this process may extend survival, it also gradually disrupts cellular homeostasis and leads to cellular dysfunction (Ramdzan et al., 2017) (Fig. 1).

### 2.2 Impairment of autophagy in HD and Htt loss of function

Disruptions in autophagy are thought to contribute to the pathogenesis of HD (Martin et al., 2015). The effects of mHtt along the autophagic pathway are multiple and pleiotropic, as mHtt interferes with processes such as autophagosomal dynamics and initiation of autophagy (Martin et al., 2015). Mechanistically, mHtt may disturb or fail to mimic the physiological roles of wild-type Htt in regulating autophagosomal transport (Wong and Holzbaur, 2014), autophagosomal cargo loading, or the activity and levels of key initiators of the autophagy pathway, such as ULK1 and Beclin-1 (Rui et al., 2015a; Wold et al., 2016; Ashkenazi et al., 2017), as detailed below.

Data from HD cell models where autophagosomes contained little cytosolic cargo suggested that mHtt may impair cargo recognition (Martinez-Vicente et al., 2010). Alternatively, studies in neurons suggested that mHtt impairs the autophagosomal transport regulation exerted by wild-type Htt and its associated protein HAP-1 (Wong and Holzbaur, 2014). Consequently, mHtt disrupts the axonal transport of autophagosomes, without altering their formation or cargo loading, but reducing autophagosome-lysosome fusion events, thereby leading to insufficient lysosomal acidification and impaired degradation of cargo (Wong and Holzbaur, 2014).

Fibroblasts from HD patients showed reduced levels of a key initiator of autophagy – beclin-1 (Ashkenazi et al., 2017). The authors proposed that mHtt competes with the deubiquitinase ataxin-3 for beclin-1 binding, thus increasing the ubiquitination and proteasomal degradation of beclin-1 and thereby impairing initiation of autophagy (Ashkenazi et al., 2017). Dysfunction in the initiation of autophagy was also attributed to the loss in mHtt of a physiological function of wild-type Htt in selective autophagy (Rui et al., 2015a, b). Htt interacts with the autophagy adaptor p62, affecting its affinity for substrates and for LC3, and it also interacts with and modulates the activation of ULK1 – a regulatory kinase involved in autophagy activation. Htt may thus act as a scaffold that brings together the machinery required for cargo recognition and initiation of autophagy (Rui et al., 2015a). Supporting this hypothesis, an independent study showed a reduced activity of ULK1 and Vsp34 kinases in HD cellular and animal models (Antonioli et al., 2017; Wold et al., 2016).

The hypothesis that Htt acts as an autophagy-promoting scaffold is further supported by the high similarity between Htt and the Atg11 protein that mediates selective autophagy in yeast (Ochaba et al., 2014). Consistently, expression of full-length Htt lacking a polyQ stretch (ΔQ-Htt) enhanced neuronal autophagy in HD mice (Hdh^140Q/ΔQ^) (Zheng et al., 2010), suggesting that polyQ expansion in Htt disrupts its physiological role as an autophagy-promoting scaffold. These studies highlight the importance of selectively targeting mHtt, as treatments that non-selectively decrease the levels of both mutant and wild-type Htt might aggravate dysfunctional autophagy.

### 2.3 Mitochondrial dysfunction and impaired proteostasis in HD

Multiple abnormalities in mitochondrial bioenergetics, dynamics and quality control have been associated with HD pathogenesis (Damiano et al., 2010; Guedes-Dias et al., 2016; Oliveira, 2010). Such abnormalities contribute to energy impairment in HD, whose nature is more dynamic than previously thought, as shifts in metabolic flux occur as compensatory homeostatic mechanisms throughout the disease (for a recent review see Dubinsky, 2017). Mechanistically, mHtt is proposed to exert indirect effects on mitochondria and energy metabolism *via* transcriptional deregulation, and also direct effects by physically interacting with the organelle and associated proteins. While mitochondrial respiratory chain impairment in HD is currently considered a late, secondary event, bioenergetic efficiency may be compromised by changes in mitochondrial dynamics, as mitochondrial structural remodelling is linked with the adaptation to energetic demands (Liesa and Shirihai, 2013), and also because neuronal processes require optimal mitochondrial distribution and size adaptation (Misgeld and Schwarz, 2017).

Increased mitochondrial fragmentation has been observed in several HD models and patients’ brains and linked to dysregulation of fission and fusion proteins. mHtt directly interacts with the fission promoter Drp1 or induces its post-translational modification, enhancing its activity (Cherubini and Gines, 2017). Moreover, mHtt disrupts mitochondrial trafficking and mitophagy as aggregates may physically obstruct intracellular organellar dynamics, while diffuse mHtt may also interfere with trafficking adaptor/regulatory proteins, affecting mitochondrial and autophagosomal dynamics (Guedes-Dias et al., 2016).

The association of mHtt with mitochondria increases with age and disease progression (Orr et al., 2008; Shirendeb et al., 2011; Yano et al., 2014). mHtt was found to interact with Tim23, a component of the import machinery at the inner mitochondrial membrane (IMM), and this interaction was proposed to inhibit protein import (Yano et al., 2014). Import deficiency may be an early event in HD pathology and mediate downstream alterations in mitochondrial respiration and morphology (Franco-Iborra et al., 2018). While further studies are still necessary, the interaction of mHtt with IMM components raises the possibility that mHtt itself may be imported into the mitochondria. Interestingly, it was recently proposed that cytosolic aggregation-prone proteins can be imported into the mitochondria and degraded by mitochondrial proteases (Ruan et al., 2017). Additionally, when cytosolic proteostasis is impaired there is an accumulation of misfolded proteins in the mitochondria, which in turn induces mitochondrial stress (Ruan et al., 2017). It is thus possible that mHtt, by disrupting cytosolic proteostasis, overwhelms and impairs the mitochondrial proteolytic machinery, inducing a stress response known as the mitochondrial unfolded protein response (mtUPR). A mechanism that induces the mtUPR is the decrease in the mitochondrial protease HtrA2 (Moisoi et al., 2009). In fact, HtrA2 levels were found to be reduced in primary neurons expressing mHtt (Inagaki et al., 2008; Tagawa et al., 2007). Thus, it would be interesting to explore if the mtUPR is activated in HD and how this stress response might modulate mHtt proteostasis and neuronal survival.

A likely consequence of mHtt-induced mitochondrial dysfunction is a decrease in ATP availability, which restricts ATP-dependent activities of the proteostasis network (Hutt and Balch, 2013) and may also limit a potential hydrotrope function of ATP on protein solubility (Patel et al., 2017), thus impairing mHtt clearance and promoting its aggregation (Fig. 1). In addition to the ATP limitation, mitochondrial dysfunction may impair the autophagic component of proteostasis by other mechanisms. One possibility is that mitochondrial dysfunction disrupts ER-mitochondrial contacts, compromising autophagosome formation which seems to be enriched at those sites (Hailey et al., 2010; Hamasaki et al., 2013). Moreover, mitochondrial dysfunction may disturb the mitochondria–lysosome reciprocal regulation (Wong et al., 2018), and impair the normal lysosomal function (Demers-Lamarche et al., 2016), thus compromising the completion of autophagy.

### 2.4 Ageing and impaired proteostasis in Huntington’s disease

The age of onset in HD correlates inversely with the number of CAG repeats in the Htt gene (Finkbeiner, 2011). Still, notwithstanding rare infantile/juvenile forms of the disease, most patients express HD alleles with ∼40-50 CAG from early-life and typically become symptomatic only around mid-life (Hands et al., 2008). This delayed onset implicates biological ageing in disease manifestation, and although the detailed mechanisms remain unclear, two non-mutually exclusive hypotheses are increasingly supported by experimental evidence.

One hypothesis is that mHtt accelerates the effects of ageing on cellular function (Gasset-Rosa et al., 2017; Horvath et al., 2016). This is supported by the increased accumulation of epigenetic ageing markers in the brain of HD patients *versus* controls (Horvath et al., 2016), and by the delay of disease progression in HD mouse models submitted to strategies that extend lifespan (Sadagurski et al., 2011; Tallaksen-Greene et al., 2014). From a mechanistic perspective, the expression of mHtt in neurons exacerbates age-dependent defects on nuclear integrity and nucleocytoplasmic transport (Gasset-Rosa et al., 2017). Additionally, mHtt induces a proteotoxic stress that disrupts ER and cytosolic redox homeostasis, a pattern observed in ageing *C. elegans* and also in *C. elegans* expressing expanded polyQ protein (Kirstein et al., 2015).

Another hypothesis is that ageing challenges cellular homeostasis and renders cells more vulnerable to mHtt toxicity (Kaushik and Cuervo, 2015; Saxena and Caroni, 2011). HD animal models present age-dependent increases in mHtt aggregate load and in motor/neurodegenerative phenotypes (Diguet et al., 2009; Marcellin et al., 2012; Morley et al., 2002). Mechanistically, the decline in mitochondrial function and in proteostasis capacity may be two of the main factors contributing to increased vulnerability to mHtt toxicity with age (Labbadia and Morimoto, 2015; Sun et al., 2016). Recently, a single-cell transcriptome atlas of the *Drosophila* brain showed that the expression of OXPHOS genes declines faster during ageing in comparison to other genes, and that this decline was accompanied by decreased mitochondrial turnover (Davie et al., 2018). The accumulation of dysfunctional mitochondria decreases ATP availability and disrupts redox homeostasis (Sun et al., 2016), with a widespread cellular effect. Specifically, the resulting ATP depletion and increased reactive oxygen species (ROS) generation may impair the UPS (Segref et al., 2014) and molecular chaperones (Grunwald et al., 2014), thus compromising cellular proteostasis and increasing mHtt aggregate load (Pinho et al., in press).

The decline of proteostasis capacity with ageing is evidenced by alterations in the composition and activity of key components of the proteostasis network. Ageing human brains present decreased levels of ATP-dependent chaperones, such as Hsp70, Hsp90 or the CCT/TRiC complex (Brehme et al., 2014). Additionally, ageing *C. elegans* show reduced levels of mitochondrial chaperones and impaired activation of stress responses such as the heat shock response or the ER and mitochondrial stress responses (Dues et al., 2016; Liang et al., 2014). Interestingly, decreases in chaperones or impaired activation of stress responses in HD models correlate with increased polyQ aggregation and toxicity (Brehme et al., 2014; Labbadia et al., 2011). The importance of age-dependent proteome changes to mHtt toxicity may also be inferred from induced pluripotent stem cell (iPSCs) models. Downregulation of the ubiquitin ligase UBR5 during iPSC differentiation, or UBR5 knock-down, impaired the proteasomal degradation of mHtt, increasing mHtt aggregation and toxicity, and suggesting that a decline of UBR5 during ageing may aggravate HD pathology (Koyuncu et al., 2018).

Overall, the proteome changes that accompany ageing contribute for the proteostasis collapse in adult-onset HD, and support therapeutic strategies that aim to enhance proteostasis capacity (Fig. 1). It remains unclear, however, if the alterations of the proteome with age occur gradually or more abruptly. While the conventional ageing model proposes a gradual decline in proteostasis capacity until reaching a disease threshold, there are also alternative models supported by data in the invertebrate *C. elegans* that suggest a general or tissue-specific collapse of programmed proteostasis in early adulthood (Labbadia and Morimoto, 2015). While further research is needed, such alternative models may provide new insights into the mechanisms of selective vulnerability and adult-onset neurodegeneration.

## 3 Section II - Strategies to alleviate impaired proteostasis in HD

### 3.1 Modulating molecular chaperones

Molecular chaperones are therapeutic targets in neurodegenerative disorders, where failure in their activity contributes to the accumulation of misfolded proteins (Papsdorf and Richter, 2014; Reis et al., 2017). The levels of different chaperones were progressively reduced in brains of HD mice (Neueder et al., 2017; Yamanaka et al., 2008). Moreover, mHtt limits chaperone availability by competing with other client proteins for chaperone binding (Hipp et al., 2012), and by sequestering chaperones in mHtt aggregates (Park et al., 2013; Yu et al., 2014). Potential strategies to increase chaperone activity in HD include the activation of the heat shock response and the direct modulation of specific chaperones (Kalmar and Greensmith, 2017).

The levels of heat shock factor 1 (Hsf1 – a key activator of the heat shock response; Anckar and Sistonen, 2011) are decreased in mouse and human HD brains (Gomez-Pastor et al., 2017). The proposed mechanism is that mHtt induces the CK2a kinase and the Fbxw7 E3 ligase, which respectively phosphorylate and ubiquitinate Hsf1, thereby promoting its proteasomal degradation (Gomez-Pastor et al., 2017). Preventing Hsf1 degradation with the CK2 kinase inhibitors TID43 or emodin, or by increasing Hsf1 activity with azadiradione, reduced mHtt aggregation and attenuated disease progression in HD mice (Gomez-Pastor et al., 2017; Singh et al., 2018).

The direct modulation of specific chaperones of the Hsp70 and Hsp90 chaperone families is another potential therapeutic strategy in HD (Pratt et al., 2015; Reis et al., 2017). Hsp70 and Hsp90 are involved in mHtt triage, but while Hsp70 interacts with proteins at an early stage of folding, either assisting their folding or redirecting them for degradation, Hsp90 acts in late folding stages, stabilizing and inhibiting client protein ubiquitination (Karagoz and Rudiger, 2015; Pratt et al., 2015). Hsp90 is thought to mediate mHtt accumulation and aggregation through the recruitment of the deubiquitinase Usp19 – which deubiquitinates mHtt and thus decreases its elimination (He et al., 2017, 2016). Pharmacological inhibition of Hsp90 with NVP-AUY992 increased mHtt degradation through the UPS by inhibiting the formation of the Hsp90-mHtt complex, leaving mHtt free to become a substrate for UPS degradation (Baldo et al., 2012). Hsp90 inhibition can also elicit the heat shock response through the dissociation of the complex Hsf1-Hsp90. Hsf1 dissociation from the complex enables Hsf1 activation and consequent upregulation of several chaperones, which will attempt to correct mHtt conformation or target it for degradation (Jackrel and Shorter, 2011). Accordingly, treatment with HSP990, an Hsp90 inhibitor, increased the levels of Hsp70 and Hsp40, and transiently ameliorated motor performance and decreased mHtt aggregate load in R6/2 mice (Labbadia et al., 2011).

Overexpression of Hsp70 decreased mHtt aggregation in cellular and animal models of HD (Guzhova et al., 2011; Hay et al., 2004), whereas Hsp70 deletion increased the size of mHtt aggregates and exacerbated the mHtt induced phenotype in R6/2 mice (Wacker et al., 2009). Hsp70 acts in combination with other chaperones to mediate protein disaggregation (Nillegoda et al., 2018). Hsp70 forms a trimeric complex with Hsp40 and Hsp110 that regulates mHtt solubility through disaggregation of mHtt fibrils (Scior et al., 2018). Recent data indicate that Hsp40 is the limiting chaperone for the disaggregation activity, since increasing Hsp40 sufficed to increase the efficiency of the complex to suppress mHtt fibrilization (Scior et al., 2018).

Proteins that fail to reach proper folding states after cycling with Hsp70 can be transferred to another group of molecular chaperones called chaperonins. Chaperonins are cylindrical complexes that encapsulate and fold single protein chains (Kim et al., 2013). Experiments manipulating expression levels of one of these chaperonins −CCT (chaperonin containing TCP-1; TRiC) – suggest that CCT reduces mHtt levels and prevents its aggregation (Tam et al., 2006; Zhao et al., 2016b). The effect of CCT in mHtt proteostasis was primarily attributed to its function as a chaperonin (Tam et al., 2006), however, more recently, it has been proposed that CCT may regulate mHtt levels through indirect mechanisms involving the protein degradation pathways. In the BACHD mouse model, CCT overexpression reduced mHtt by enhancing its proteasomal degradation (Zhao et al., 2016b). In HeLa cells expressing mHtt constructs, CCT knockdown impaired the autophagic flux and increased mHtt aggregation, due to a loss of function of CCT in actin folding, disrupting the cytoskeleton, and consequently intracellular transport or autophagosomal biogenesis and degradation (Kast and Dominguez, 2017; Pavel et al., 2016). The function of CCT in actin folding may also explain why CCT overexpression was able to rescue BDNF and lysosomal transport in i*n vivo* HD models (Zhao et al., 2016b).

Collectively, in addition to modulation of Hsp70 and Hsp90, these data support the modulation of other chaperone families, such as Hsp40 and chaperonins, alone or in combination with Hsp70/90, as potential therapeutic strategies in HD.

### 3.2 Modulating the ubiquitin-proteasome system

The UPS is one of the main routes for degradation of misfolded proteins, and its impairment has been associated with several neurodegenerative diseases, including HD (McKinnon and Tabrizi, 2014). Impairment of the UPS in HD has been attributed to an inefficient targeting of soluble mHtt for proteasomal degradation (Bhat et al., 2014) and, more recently, to the inhibition of 26S proteasome gate opening by soluble oligomeric mHtt species (Thibaudeau et al., 2018). This provides a rationale to enhance UPS activity as a therapeutic strategy, and this has been tested in HD models through the modulation of proteasome subunits and of ubiquitin-interacting proteins, such as ubiquitin ligases, ubiquilins, and deubiquitinases (Eletr and Wilkinson, 2014; McKinnon and Tabrizi, 2014; Ristic et al., 2014).

Direct proteasome modulation *via* overexpression of the proteasome activator PA28ɣ increased cell viability in HD neuronal models (Seo et al., 2007). Subsequent *in vivo* studies showed that PA28ɣ overexpression rescued HD phenotypes in YAC128 mice (Jeon et al., 2016), whereas overexpression of the proteasomal pbs-5 catalytic subunit increased resistance to proteotoxic stress and ameliorated motor phenotypes in HD nematodes (Chondrogianni et al., 2015).

**Fig. 2:**
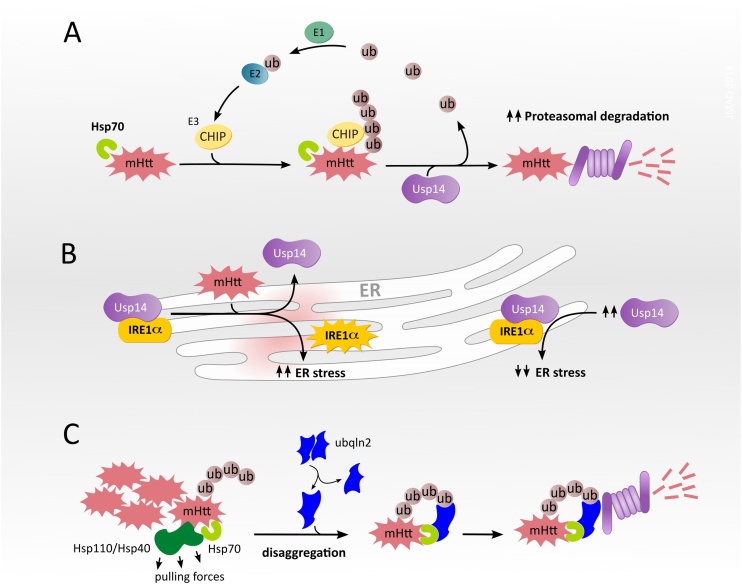
**Modulation of the UPS in HD. (A)** The E3 ubiquitin ligase CHIP polyubiquitinates mHtt bound to Hsp70, targeting mHtt for proteasomal degradation (Jana et al., 2005; Kettern et al., 2010; Reeg et al., 2016). The deubiquitinase Usp14 removes the ubiquitin (ub) chains and increases the accessibility of mHtt for proteasomal degradation (Hyrskyluoto et al., 2014). Premature deubiquitination by Usp14 may allow substrates to escape degradation (Lee et al., 2010, 2016), see section “*Modulating the ubiquitin-proteasome system”* for details. **(B)** Left: mHtt decreases the interaction between Usp14 and the ER stress transducer IRE1α, enhancing phosphorylation and activation of IRE1α. Right: increased expression of Usp14 limits IRE1α activation, reducing mHtt-induced ER stress (Hyrskyluoto et al., 2014). **(C)** Ubiquilin 2 (ubqln2) recognizes mHtt aggregates bound to the disaggregase complex Hsp70-Hsp40-Hsp110. Following disaggregation, ubiquilin 2 shuttles mHtt to the proteasome, promoting its degradation (Hjerpe et al., 2016).

The overexpression of E3 ubiquitin ligases such as CHIP, Ube3, Herp or UBR5, is a potential strategy to decrease mHtt aggregation, by promoting its ubiquitination and proteasomal degradation (Bhat et al., 2014; Jana et al., 2005; Luo et al., 2018) (Koyuncu et al., 2018). The related strategy of overexpressing proteins that recruit ubiquitin ligases, such as NUB1 (negative regulator of ubiquitin-like protein 1), reduced mHtt neurotoxicity in flies (Aron et al., 2013; Lu et al., 2013). Alternatively, one might target proteasomal shuttles, such as ubiquilin-2, which cooperates with the Hsp70-Hsp110 disaggregase machinery, and mediates the delivery of Hsp70-bound mHtt to the proteasome for degradation (Hjerpe et al., 2016) (Fig. 2).

Inhibition of the proteasome-associated deubiquitinase Usp14, with the small molecule inhibitor IU1, decreased levels of disease-associated proteins in cell models of the polyQ disorder SCA3, Alzheimer′s disease, amyotrophic lateral sclerosis, and Prion disease (Lee et al., 2010; McKinnon et al., 2016). The authors proposed that a premature deubiquitination of the substrates by Usp14 allows them to escape proteasomal degradation, and thus inhibiting Usp14 with IU1 increases substrate degradation (Lee et al., 2010, 2016). We found no published studies testing this Usp14 inhibition strategy in HD models. In contrast, Usp14 overexpression reduced aggregates and death in cells expressing mHtt (Hyrskyluoto et al., 2014).

Unlike the premature deubiquitination hypothesis, the Usp14 overexpression strategy aims to deubiquitinate mHtt aggregates making them more accessible to proteasomal degradation (Hyrskyluoto et al., 2014). Additionally, Usp14 overexpression inhibited IRE1α phosphorylation, thus preventing mHtt from inducing an ER stress pathway that impairs autophagy and increases mHtt accumulation (Hyrskyluoto et al., 2014; Lee et al., 2012). While further studies are necessary to clarify the role of the deubiquitinase Usp14 in mHtt proteostasis, current data suggest it plays a dual role, regulating accessibility of ubiquitinated mHtt to the UPS, and also limiting mHtt-induced ER stress (Fig. 2).

Enhancement of the UPS activity has the potential to decrease mHtt soluble levels, mHtt aggregation and its induced cytotoxicity. While genetic strategies have thus far been the predominant approach to modulate the UPS, pharmacological investigations are likely to increase with the recent development of chemical modulators of the UPS pathway (Collins et al., 2017; Khan and Nelson, 2018; Leestemaker et al., 2017).

### 3.3 Modulating autophagy

**Fig. 3:**
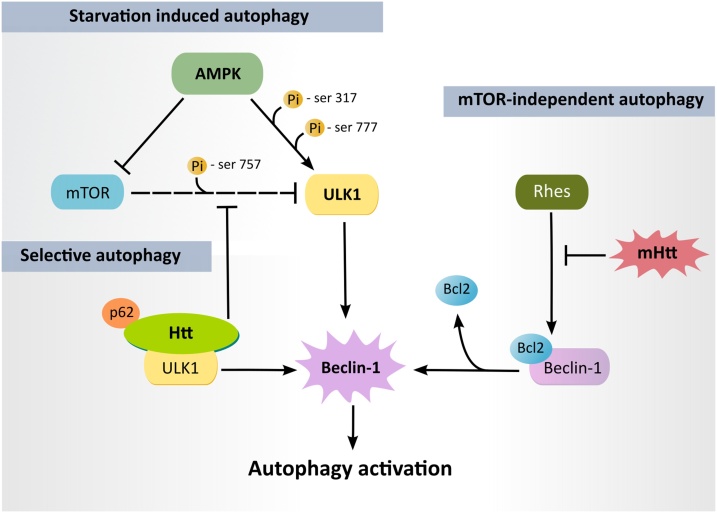
**Modulation of autophagy in HD.** In *starvation induced autophagy* (nutrient and energy depletion), AMPK activates ULK1 (*via* its phosphorylation at serines 317 and 777) and inhibits mTOR, preventing its inhibitory phosphorylation of ULK1 at serine 757 (Kim et al., 2011). In *mTOR-independent autophagy*, AMPK phosphorylates ULK1 without affecting mTOR activity (Walter et al., 2016). Rhes may also induce mTOR-independent autophagy by releasing Beclin-1 from the inhibitory effect of Bcl2 (Mealer et al., 2014). mHtt may block this effect of Rhes, contributing to autophagy impairment in HD (Mealer et al., 2014). In *selective autophagy*, wild-type Htt may act as a scaffold that bridges autophagy recognition and activating machinery (p62 and ULK1, respectively), and releases ULK1 from the inhibitory effect of mTOR (Rui et al., 2015a). Active ULK1 phosphorylates Beclin-1, leading to autophagy activation.

Autophagy is regulated through pathways that sense the cellular energy status, nutrient supply, and the availability of growth factors. The initial step of autophagosome formation is primarily mediated by the ULK1 complex, whose activity is regulated by two major upstream kinases: mTORC1 (mammalian target of rapamycin – mTOR, complex 1) and AMPK (AMP activated kinase) (Hurley and Young, 2017). Under conditions of nutrient availability, mTORC1 inhibits ULK1 by phosphorylation at Ser 757, suppressing autophagosome formation. Under conditions of nutrient deprivation (starvation), AMPK activates ULK1 directly *via* phosphorylation at Ser 317 and Ser 777, and indirectly through inhibition of mTORC1 (Kim et al., 2011) (Fig. 3). The modulation of mTORC1 and AMPK activities can thus regulate autophagy and, to date, these have been two of the most extensively studied strategies to modulate autophagy in HD models (Crino, 2016; Vingtdeux et al., 2011).

Despite earlier indications that the inhibition of mTORC1 has beneficial effects in HD models (Ravikumar et al., 2004; Roscic et al., 2011), more recently, the restoration of mTORC1 activity was shown to improve motor deficits and brain pathology in HD mice (Lee et al., 2015). mTORC1 activity was reduced in the striatum of HD patients and also in the striatum of N171-81Q mice (Lee et al., 2015). Moreover, in N171-81Q mice, the expression of the constitutively active form of the mTORC1 activator Rheb (Ras homolog enriched in brain) upregulated basal autophagy and increased mHtt clearance (Lee et al., 2015). Additionally, disease phenotypes were also rescued in N171-81Q mice by exogenous expression of Rhes (Ras homolog enriched in striatum; (Lee et al., 2015). Nevertheless, the precise role of mTORC1 activity and Rhes in HD pathology remains controversial.

Rhes was shown to interact with mHtt and promote its cytotoxicity (Subramaniam et al., 2009). Rhes deletion improved disease progression in HD mice (Baiamonte et al., 2013; Mealer et al., 2013; Swarnkar et al., 2015), whereas ectopic Rhes expression in the cerebellum of HD mice exacerbated the disease phenotype (Swarnkar et al., 2015). There is evidence that Rhes promotes autophagy in cells, but in an mTORC1-independent manner by binding to beclin-1 and reducing its inhibitory interaction with Bcl-2 (Mealer et al., 2014). The interaction with mHtt seems to block such autophagy-promoting activity of Rhes, thus contributing to impairment of autophagy (Mealer et al., 2014) (Fig. 3).

The modulation of mTOR-independent autophagy has shown promising results in HD models. Pharmacological activation or expression of constitutively active AMPK induced autophagy in an mTOR-independent manner, reduced mHtt aggregates and improved cell viability (Walter et al., 2016). Pharmacological induction of autophagy with the Akt inhibitor 10-NCP reduced mHtt levels and improved survival of striatal neurons (Tsvetkov et al., 2010), while inhibition of the alpha-tubulin deacetylase HDAC6 with tubastatin A promoted autophagic flux and decreased mHtt levels in striatal neurons (Guedes-Dias et al., 2015). Despite the encouraging results of indirectly modulating autophagy by targeting mTOR-dependent and mTOR-independent pathways, there has recently been an increased effort to identify strategies to modulate autophagy more directly. One such strategy is regulating the activity of the autophagy initiation complex ULK1.

The activity of ULK1 seems to be decreased in the brains of zQ175 HD mice, as suggested by the decreased phosphorylation of ULK1 substrates Beclin-1 and Atg14, together with the redistribution of ULK1 to an insoluble fraction where aggregated mHtt was found (Wold et al., 2016). In turn, overexpression of wild-type ULK1 but not that of a kinase inactive form of ULK1, decreased insoluble mHtt levels in cell lines, suggesting that ULK1 kinase activity is a limiting factor for the autophagic clearance of mHtt (Wold et al., 2016). The direct modulation of ULK1 activity thus shows potential to modify mHtt load, an exciting hypothesis that is now possible to test with the recent development of ULK1 activators (Zhang et al., 2017) and ULK1 inhibitors (Egan et al., 2015; Lazarus et al., 2015; Lazarus and Shokat, 2015; Petherick et al., 2015).

Lastly, there is increased evidence that mHtt clearance may be also regulated by chaperone-mediated autophagy (CMA; Bauer et al., 2010; Koga et al., 2011; Qi et al., 2012; Thompson et al., 2009). These findings have promoted the search for small molecule inducers of CMA. Given that signalling *via* the retinoic acid receptor α (RARα) seems to inhibit CMA, RARα antagonists are being investigated as chemical enhancers of CMA to tackle proteotoxicity in neurodegenerative disorders and ageing (Anguiano et al., 2013). Still, as several components involved in autophagy and CMA decline with age (Kaushik and Cuervo, 2015), complementary strategies to modulate the Htt proteostasis network in HD are being actively pursued, namely the modulation of Htt post translational modifications.

### 3.4 Modulating huntingtin post translational modifications

Post translational modifications (PTMs) are key modulators of Htt conformation, regulating its interaction with other proteins, its stability, aggregation, subcellular localization and clearance (Ehrnhoefer et al., 2011). The presence of expanded polyQ significantly alters PTMs. Among them, proteolytic cleavage, phosphorylation, acetylation, ubiquitination, and SUMOylation have all been shown to alter Htt proteostasis, and modulation of each of these represents a potential therapeutic strategy.

Proteolytic cleavage of mHtt yields toxic N-terminal fragments containing expanded polyQ (Ehrnhoefer et al., 2011). The activity of the protease caspase 6 is increased in HD (Graham et al., 2010), possibly because mHtt hinders the normal inhibitory binding of wild-type Htt to the proform of caspase 6 (Riechers et al., 2016). Caspase 6 cleaves Htt at the amino acid 586. Expression of the resulting mHtt-586 N-terminal fragment aggravates the phenotype of HD mice (Waldron-Roby et al., 2012), whereas a 586 cleavage-resistant mutant or caspase 6 knockout attenuates the phenotype (Gafni et al., 2012; Graham et al., 2006; Pouladi et al., 2009; Wong et al., 2015). While these studies highlight the toxicity of N-terminal fragments, C-terminal fragments have also been proposed to play a role in HD pathophysiology. The presence of an Htt C-terminal fragment in *in vitro* and *in vivo* HD models induced ER toxicity, possibly through a mechanism that inhibits dynamin-1, a GTPase involved in endocytic membrane fusion and recently found in the ER (El-Daher et al., 2015). Still, further studies are necessary to clarify the differential proteostasis and toxicity of the Htt C-terminal fragment in wild-type *versus* mHtt-expressing cells.

The Htt N-terminal has multiple phosphorylation sites that regulate Htt conformation, its interaction with other proteins, and also its proteolytic accessibility (Guo et al., 2018). Mutant Htt is less phosphorylated than the wild-type form, and increased phosphorylation at specific residues decreases mHtt toxicity (Thompson et al., 2009). The first 17 acids contain a cytoplasmic retention signal (CRS) and three phosphorylation residues (T3, S13, and S16) (Ehrnhoefer et al., 2011). S13 and S16 phosphorylation alter the CRS, increasing mHtt nuclear localization (Atwal et al., 2011; Thompson et al., 2009) and reducing neuronal death (Arbez et al., 2017). S421 phosphorylation increased mHtt proteasomal degradation and rescued neurodegeneration in HD mice (Kratter et al., 2016). Interestingly, the phospho-null S1201 A mutation reduced mHtt neurotoxicity (Arbez et al., 2017), while also increasing its nuclear localization (Ratovitski et al., 2017), which may tag mHtt for proteasomal and lysosomal degradation (Greiner and Yang, 2011; Thompson et al., 2009). Concerning mHtt conformation and aggregation, S13 and S16 phosphorylation reduced the mHtt conformational rigidity caused by the polyQ expansion (Daldin et al., 2017) and decreased its aggregation in human cells (Branco-Santos et al., 2017). Similarly, increasing mHtt T3 phosphorylation, which is strongly decreased in HD mice and human cells, reduced its conformational rigidity (Cariulo et al., 2017; Chiki et al., 2017) and reduced aggregation in HD flies (Branco-Santos et al., 2017).

In contrast to phosphorylation, mHtt is preferentially acetylated compared to the wild-type form (Ehrnhoefer et al., 2011). Acetylation at lysine K6 reversed the inhibitory effect that T3 phosphorylation had on mHtt aggregation *in vitro* (Chiki et al., 2017). K444 acetylation was found in mutant, but not wild-type, Htt, and was proposed to facilitate trafficking of mHtt into autophagosomes, increasing its autophagic clearance and reducing its toxic effects in primary neurons and in a *C. elegans* HD model (Jeong et al., 2009).

Htt ubiquitination, particularly at lysines K6, K9 and K15, increases its degradation (Martin et al., 2015). In contrast, SUMOylation – the attachment of small-ubiquitin-like modifiers (SUMO), competes with ubiquitination, promoting mHtt stability and reduced aggregation, which increases its potential to exert toxic effects (Steffan et al., 2004). The knockout of E3-SUMO ligases improved disease phenotypes in HD mice. In particular, PIAS1 knockdown in HD mice decreased mHtt inclusions, the accumulation of ubiquitinated and SUMOylated proteins, and also ameliorated the inflammatory and behavioural phenotypes (Ochaba et al., 2016).

### 3.5 Modulating mitochondria

Given the several pathways by which mitochondrial dysfunction may impair cellular proteostasis, one potential strategy to restore proteostasis in neurodegenerative disorders might be to rescue mitochondrial function. The modulation of mitochondrial metabolism and bioenergetics is under active research in this context (Sorrentino et al., 2018). Treatment with MSDC-0160, an inhibitor of the mitochondrial pyruvate carrier, ameliorated the pathology in cell and mammalian models of Parkinson′s disease, through a mechanism involving inhibition of mTOR and induction of autophagy (Ghosh et al., 2016). In cellular and mouse models of HD, treatment with butyrate or phenylbutyrate enhanced pyruvate dehydrogenase activity and this was associated with a rescue of mitochondrial metabolic function and motor phenotypes (Naia et al., 2017a). Also, treatment with resveratrol was linked with sirtuin 1 activation and a rescue of mitochondrial function in cellular HD models and motor phenotypes in YAC128 HD mice (Naia et al., 2017b). Still, given the pleiotropic effects of resveratrol on mitochondria, further studies are warranted to understand its metabolic targets (Sorrentino et al., 2018).

The rescue of mitochondrial function by decreasing oxidative stress or by modulating mitochondrial dynamics has also been tested in HD models (Damiano et al., 2010). Treatment of the ST*Hdh*^Q111/Q111^ cell line with the mitochondria-targeted antioxidant MitoQ was found to alter the expression of genes involved in mitochondrial fission-fusion dynamics (Yin et al., 2016). In mouse models of other neurodegenerative disorders, such as Alzheimer’s disease and spinocerebellar ataxia type I, treatment with of MitoQ was found to rescue mitochondrial impairments and disease phenotypes (McManus et al., 2011). In HD cell line models, treatment with the compound mdivi-1 promoted mitochondrial fusion, an effect that was associated with the inhibition of excessive mitochondrial fission (Manczak and Reddy, 2015), although this mechanism of mdivi-1 activity has been recently questioned and instead attributed to the modulation of mitochondrial ROS production (Bordt et al., 2017).

The enhancement of mitochondrial proteostasis mechanisms is currently being explored as a means to alleviate the cytosolic proteostasis machinery and reduce the proteotoxicity of misfolded proteins (Ruan et al., 2017). The genetic and pharmacological enhancement of mitochondrial proteostasis, in a *C. elegans* model of amyloid-β (Aβ) proteotoxicity, decreased Aβ aggregation and Aβ-induced toxicity (Sorrentino et al., 2017). In *C. elegan*s expressing mHtt, the induction of mitochondrial proteostasis, by mild perturbation of the electron transport chain, reduced mHtt-induced toxicity and polyQ aggregation with age (Kim et al., 2016; Labbadia et al., 2017). Similar results were observed when mitochondrial proteostasis was induced by reduction of mitochondrial Hsp70 levels or by inhibition of fatty acid import (Kim et al., 2016). Interestingly, in this case, the induction of mitochondrial proteostasis was also accompanied by an induction of the cytosolic heat shock response, suggesting that mitochondria can signal cytosolic proteostasis as an attempt to maintain cellular homeostasis (Kim et al., 2016). Overall, these findings support the modulation of mitochondrial proteostasis as an avenue to recover impaired proteostasis in HD, linking mitochondrial (dys)function with cytosolic stress responses.

## 4 Concluding remarks

The current model to account for the impairment of proteostasis by mHtt proposes a gradual, yet global collapse of the proteostasis network that synergises with ageing-associated changes in proteostasis capacity. Molecular chaperones, proteolytic pathways such as the UPS and autophagy, and Htt PTMs are among the most intensively tested therapeutic targets. Studies on the role of the RQC system for mHtt proteostasis, and also of the impact of the unfolded protein responses of the ER and mitochondria upon cellular proteostasis, are opening new possibilities for experimental therapeutics.

A long standing question in HD research is how the ubiquitously expressed mHtt preferentially kills neurons, particularly medium spiny striatal neurons (Margulis and Finkbeiner, 2014). Intrinsic differences in the proteostasis system among cellular populations may contribute to this differential vulnerability. Consistently, the clearance of soluble mHtt seems slower in neurons than in astrocytes, and also slower in striatal than in cortical neurons (Zhao et al., 2016a). This differential mHtt proteostasis was attributed to a preferential clearance of mHtt by the UPS (Zhao et al., 2016a). Accordingly, UPS activity seems higher in astrocytes than neurons (Tydlacka et al., 2008), and cortical neurons expressing mHtt present a higher dependence on the UPS than striatal neurons (Tsvetkov et al., 2013). These findings support the enhancement of UPS activity to reduce neuronal vulnerability in HD.

A more recent question in the HD field is whether Htt acts as a scaffold in the autophagy process. This hypothesis is now further supported by evidence that Htt interacts with autophagy inducers such as ULK1 (Rui et al., 2015a), whose activity was recently found reduced in HD models (Wold et al., 2016), possibly contributing to autophagy impairment in HD. Importantly, ULK1 modulators are emerging in the context of cancer research (Egan et al., 2015; Zhang et al., 2017) and it will be interesting to evaluate their potential in the context of protein-misfolding diseases.

In addition to directly targeting the UPS and autophagy pathways, the clearance of mHtt may also be regulated by modulating molecular chaperones. Thus far, the most common approaches in the context of HD have been Hsp70 and Hsp90 modulation (Reis et al., 2017). However, the modulation of other chaperones, such as Hsp40, which cooperates with Hsp70 to mediate mHtt disaggregation (Scior et al., 2018), and chaperonins like CCT, which may intervene when Hsp70 folding activity fails and prevent mHtt aggregation (Kim et al., 2013; Tam et al., 2006), arise as potential therapeutic strategies in HD.

Other attempts to enhance cytosolic proteostasis may involve the modulation of the unfolded protein response of organelles such as the ER or mitochondria (Rahman et al., 2018; Sorrentino et al., 2017). Regarding the ER, many neurodegenerative diseases, including HD, have been associated with chronic activation of the ER stress response (Hyrskyluoto et al., 2014; Remondelli and Renna, 2017; Zhou et al., 2018). Increasing the ER folding capacity may thus be beneficial for HD treatment. Accordingly, modulation of protein disulphide isomerase, an ER chaperone, increased the life span and ameliorated motor dysfunction and brain atrophy in HD mice (Zhou et al., 2018). Regarding mitochondria, this organelle was recently proposed as a site for degradation of cytosolic aggregates (Ruan et al., 2017). Moreover, enhancing mitochondrial proteostasis reduced mHtt aggregation and toxicity (Labbadia et al., 2017). Mitochondria contain their own set of chaperones and proteostasis machinery with the potential of being modulated (Braun and Westermann, 2017). Inhibitors of mitochondrial proteases are being developed, however, they still require improvements in specificity and further characterization of their mechanism of action (Goard and Schimmer, 2014).

HD thus far remains an incurable disease without effective treatment. Nevertheless, there is a growing understanding of the interaction between the different elements of the proteostasis network and different mHtt species. This may soon provide an integrated view of how differential proteostasis influences neuronal vulnerability. New molecules targeting different elements of the proteostasis network are currently emerging, bringing exciting prospects for the future pharmacological management of HD and related disorders.

## Conflict of interest

The authors declare they have no conflict of interest.
